# Lead Poisoning-Induced Hypertensive Crisis Managed by Prazosin: A Case Report

**DOI:** 10.5812/ircmj.4557

**Published:** 2013-06-05

**Authors:** Bita Dadpour, Omid Mehrpour, Leila Etemad, Mohammad Moshiri

**Affiliations:** 1 Imam Reza Hospital, Mashhad University of Medical Sciences, Mashhad, IR Iran; 2 Addiction Research Centre, Mashhad University of Medial Toxicology, Mashhad, IR Iran; 3 Birjand Atherosclerosis and Coronary Artery Research Center, Birjand University of Medical Science, Birjand, IR Iran; 4 Department of Pharmacodynamy and Toxicology, School of Pharmacy, Mashhad University of Medical Sciences, Mashhad, IR Iran

**Keywords:** Lead, Hypertension, Prazosin

## Abstract

**Introduction:**

Chronic lead exposure is known to be a risk factor for hypertension (HTN). No specific medication is recommended for the treatment of lead-induced hypertension (LIHTN).

**Case Presentation:**

Our patient was a male admitted with the chief complaint of chronic abdominal pain. His whole blood lead level was reported to be 1961 µg/L. He also mentioned a previous history of HTN managed by propranolol (10 mg, TDS). He discharged himself by giving written consent and 19 days later, he was re-admitted due to high blood pressure of 220/140 mmHg. His Blood pressure (BP) was decreased to 180/110 mmHg with sublingual captopril; but, in maintenance therapy, higher doses of captopril could not further decrease BP. Amlodipine was tried which was discontinued due to the patient intolerance. Prazosin was then administered in gradual increasing doses up to 1 mg twice a day and captopril was tapered.

**Conclusions:**

We would like to suggest that LIHTN may better be managed by alpha blockers compared with converting enzyme inhibitors

## 1. Introduction

Lead toxicity is a well-known poisoning since old days. Lead effects on several organs including nervous, cardiovascular, renal, endocrine and hematologic systems (1, 2). Several studies have proven the relationship between body lead content and increasing blood pressure (BP) and also chronic lead exposure is known to be a risk factor for hypertension (HTN) (3). We report a case of lead-induced high BP which was successfully managed by prazosin. This is probably the first report recommending administration of an alpha blocker for the management of lead-induced HTN.

## 2. Case presentation

A 57-year-old man referred to us with chief complaint of chronic constant abdominal pain since one month earlier. He also suffered anorexia, constipation and bloating. On his past history, he mentioned oral opium abuse for almost 15 years. History of HTN and ischemic heart disease was present which had been controlled by administration of propranolol (10 mg, TDS) and sublingual pearl of TNG. During evaluation of his abdominal pain, duodenal ulcer and single tubular rectal polyp were also detected. On admission, his whole blood lead level was reported to be 1961 µg/L and other laboratory tests were as follow: haemoglobin = 10.9 g/dL, plasma creatinine = 1 mg/dL, blood urea nitrogen = 97 mg/dL, aspartate aminotransferase = 63 IU/L, alanine transaminase = 78 IU/L, Alkaline phosphatase = 321 IU/L, serum sodium = 133 mEq/L, and serum potassium = 4.5 mEq/L.

His opium was switched to methadone at dose of 20 mg/day and then tapered. He was treated by EDTA and BAL with the diagnosis of lead poisoning, but the chelating therapy was discontinued because he discharged himself by giving consent form. Nineteen days later, he was re-admitted to our hospital with chest pain without vomiting and diaphoresis. His blood pressure was 220/140 mmHg. After sublingual administration of 50 mg of captopril (two 25-mg tablets) his blood pressure dropped to 180/110 mmHg. On electrocardiography, he had normal axis, sinus rate (86 beats/min), and ST depression and inverted T wave in leads V4-V6. However, cardiac enzymes were within normal limits. He received oral captopril with the dose of 25 mg TDS as well as ASA 80 mg daily. Since his blood pressure did not further decrease, we increased the captopril dosage to 50 mg BID and then added another 25 mg of captopril and 5 mg of amlodipine every day. The patient began to suffer headaches and therefore, amlodipine was discontinued. This time, daily dose of 0.5 mg of prazosin was added to his medications. The response was good and during the next four days, the dosage of prazosin was gradually increased to 1 mg twice a day and captopril was simultaneously tapered. He was discharged with prazosin 1 mg twice a day and blood pressure of 140/70 mmHg. The informed consent was taken from the patient to publish the data on his disease. The ethical committee of Imam Reza hospital approved this report, as well.

## 3. Discussion

Lead is not an essential metal for human physiology and interferes with several cell metabolisms (1). The sources of lead exposure vary in each part of the world. Occupational lead exposure is an important health issue in Iran. Moreover, lead processing industry has always been a major concern since it affects surface water, drinking water, and even the Caspian Sea, the Persian Gulf and the rivers. Meanwhile, lead contamination of air and soil especially in the neighboring of the polluted and industrialized cities is another health issue in Iran. Even foods such as fish, rice, milk, and vegetables- which are the most common food of Iranian people- are contaminated with lead (4, 5). Adulteration of opium with lead and thalium is also widespread in our country (5-7). Based on our previous cases in Mashhad (7), we assumed that the only source of lead exposure in our case was opium abuse. There are many papers published about adverse effect of lead on human organs.Moreover, the relationship between blood lead levels and blood pressure has previously been established by several studies. In a 4-year follow-up study (1997–2001) of Korean lead workers, an annual rise of 10 μg/dL in blood lead levels was associated with 0.9 mmHg rise in systolic blood pressure (8).

LIHTN may be developed by several mechanisms including I: nitric oxide deficiency due to inactivation of endogenous nitric oxide by reactive oxygen species (9); II: increasing activity of sympathetic and circulating noradrenaline joined with decreasing vascular β adrenergic receptor density (10); and/or III: increasing angiotensin converting enzyme (ACE) activity and elevating rennin, angiotensin II, and aldosterone levels of plasma (11). Elevated plasma renin activity is found after periods of modest exposure although activity may decrease to normal or lower in chronic severe exposure.(12) Other mechanisms include heightening of the kininase I and kininase II activities, possible increases in endothelin and thromboxane production, and rise in cellular Na+ and hence Ca2+ stores of vascular smooth muscles due to lead inhibition of Na–K+ ATPase (13). We would like to suggest that chronic lead poisoning was a reason of HTN in this case. When he came back with chest pain, his blood pressure decreased by sublingual captopril and TNG IV drips. But maintenance therapy with captopril could not further decrease blood pressure. According to what mentioned above, although the activity of rennin system may increase at the early stage of LIHTN, it my decrease to normal level over the time (12). This may be the reason for inadequate response to captopril, an angiotensin-converting enzyme inhibitor. One of the most important mechanisms of LIHTN is intracellular Ca2+ increase secondary to Na–K+ ATPase inhibition. We, therefore, added amlodipine, a calcium channel blocker, to his medications which was later discontinued due to adverse effects and patient's intolerance.

On the other hand, lead not only could increase the activity of sympathetic and circulatory noradrenaline- a powerful vasoconstrictor- but also might decrease the vascular β, e.g. the effective arterial vasodilator. It therefore seems that one of the important mechanisms of LIHTN is the increase in the vascular tone. According to this hypothesis, we changed captopril to prazosin- an alpha adrenergic blocker- with the dose of 1 mg BID, which successfully managed the patient's HTN. In conclusion, we would like to suggest that accelerated HTN due to lead toxicity better responds to alpha blockers compared with converting enzyme inhibitors; however, this hypothesis needs to be further evaluated in future studies.
